# Cognitive dysfunction in diabetes-related foot complications: A cohort study

**DOI:** 10.1007/s40200-023-01381-4

**Published:** 2024-01-22

**Authors:** Mai Loan Nguyen, Dana Wong, Elizabeth Barson, Eva Staunton, Caroline A. Fisher

**Affiliations:** 1 School of Psychology and Public Health, La Trobe University, Bundoora, VIC 3086 Australia; 2 Psychosocial Oncology Program, Peter MacCallum Cancer Centre, Grattan Street, Parkville Victoria, 3052 Australia; 3 Allied Health – Podiatry, The Royal Melbourne Hospital, Grattan Street, Parkville Victoria, 3052 Australia; 4 Allied Health – Psychology, 4 North, The Royal Melbourne Hospital, 300 Grattan Street, Parkville Victoria, 3052 Australia; 5 The Melbourne Clinic, 130 Church St, Richmond Victorian, 3121 Australia

**Keywords:** Ulcer, Memory, Executive function, Self-management, Vascular disease, Glycated haemoglobin

## Abstract

**Objective:**

Mild-moderate cognitive impairment has been identified in general diabetes, and early evidence indicates cognitive reductions may be more pronounced in those with diabetes-related foot complications (DRFC). Cognitive difficulties may impede treatment engagement and self-management. This requires further explication to optimise patient care and outcomes. The current study aimed to characterise cognitive function in people with DRFC using comprehensive cognitive measures.

**Method:**

This cross-sectional cohort study recruited 80 adult participants (*M*_*age*_ = 63.38, SD = 11.40, range = 30 – 89) from the Royal Melbourne Hospital Diabetic Foot Unit in Victoria, Australia, all with DRFC. Each completed a comprehensive cognitive battery (memory, attention, executive functions) and scores were calculated using age-matched population norms, where available.

**Results:**

On the majority of tasks, DRFC participants performed significantly worse than age-matched norms, with the largest decrements seen in inhibition control, verbal memory, verbal abstract reasoning and working memory. Small to moderate reductions were also seen in visual learning, verbal fluency, processing speed and premorbid functioning. Demographic (lower education, male gender) and clinical factors (higher HbA1c, macrovascular and microvascular disease, longer diabetes duration) were associated with poorer cognitive functioning.

**Conclusions:**

Marked reductions in cognitive functioning were found in individuals with DRFC, predominantly in the domains of verbal memory and executive functioning. Lower education, male gender and indicators of diabetes severity, such as vascular disease, are associated with heightened risk for poorer cognitive functioning. As DRFCs are a serious complication with devastating outcomes if not successfully managed, cognitive barriers to self-management must be addressed to optimise treatment.

**Supplementary Information:**

The online version contains supplementary material available at 10.1007/s40200-023-01381-4.

## Introduction

Diabetes-related foot complications (DRFC) are serious sequelae that require ongoing self-management. This often includes self-inspections of the foot, continued engagement with health services, specific prescribed footwear and appropriate activity levels [1]. Inadequate self-management may contribute amputation rates and excess mortality (56.6% in five years [2, 3]. Yet, the cognitive barriers to self-management are not well understood. While the cognitive sequelae of DM have been previously documented [4], less is known, specifically, about cognition in those with DRFC. It is crucial to characterise and better understand the cognitive challenges of this specific DM subgroup in order to inform service design and improve patient care.

Although mild-moderate cognitive impairment has already been identified in DM [4, 5], the additional burden of a DRFC may be associated with more severe cognitive impairment. Compared to the general DM population, individuals with DRFC have a longer diabetes duration, lower body mass index and creatinine clearance [6]. Higher rates of coronary artery disease, retinopathy, nephropathy and arterial disease in the lower extremities, have also been found in those with DRFC [6]. This may indicate increased risk of cerebral alterations and correspondingly poorer cognitive outcomes [7]. Natovich et al. [8] reported that individuals with DRFC performed poorer in all cognitive domains assessed (executive function, attention, psychomotor ability and memory) in relation to those with DM without foot complications, with comparable results found only on a test estimating premorbid cognitive functioning. Similarly, global cognitive impairment has been associated with DRFCs when using the Mini-Mental State Examination [9]. Nevertheless, the existing literature pertaining to cognition in the DRFC population has not been comprehensive. Two studies have only utilised brief cognitive screening measures [9, 10]. One study has used a small range of subtests, focusing on discrete cognitive areas [11]. The fourth study used a wide-ranging assessment battery; however, this battery is administered online and has not been widely investigated in the literature or employed in general clinical practice [8].

While brief cognitive assessments and screens are useful to promptly identify individuals at risk for cognitive impairment, the domains assessed are narrow in range and provide limited scope for clinical translation [12]. In comparison, neuropsychological assessments comprehensively examine individuals’ functioning across numerous domains, allowing for individualised treatment planning. Specifically, it is necessary to separately assess discrete cognitive functions that play different roles in self-management. For example, problems with learning and memory may cause difficulty with medication adherence, and with the appropriate use of glucose monitoring and insulin devices [13]. Attentional problems can hinder encoding of new information, such as when management plans change, and further compound memory dysfunction [14, 15]. Reduced planning abilities can impede meal planning and preparation, scheduling and tracking appointments, and incorporating physical activity into daily routines [13, 16].

### Our study

Our study builds on two recently conducted DRFC psychological studies. The first investigated performance on cognitive screening assessment and a health beliefs questionnaire [10]. The average score on cognitive screening was below general population age-matched means, with a quarter of scores in line with early dementia samples [10]. A second study [17] conducted interviews with experienced multidisciplinary DRFC clinicians investigating their views on the psychological and cognitive functioning of their DRFC client cohort. Responses indicated that global cognitive dysfunction (memory, executive function, comprehension of health information, insight) influenced treatment efficacy and self-management for DRFC patients.

We aimed to elucidate cognitive issues that may be present in individuals with DRFC using a comprehensive assessment battery. Additionally, we aimed to investigate the demographic and clinical variables associated with each domain of cognitive dysfunction, to provide insight about risk factors for specific cognitive difficulties and guide clinical recommendations. We aimed to provide a clinically applicable evidence base for clinical neuropsychologists working with this patient group. A better understanding of cognitive functioning in DRFC will help medical and allied health care teams determine when, and with whom, neuropsychology input may be most useful. Finally, results from this project will also inform the design of a neuropsychological intervention aimed at addressing cognitive barriers to treatment adherence and self-management.

#### Research aims

1. To assess the neuropsychological profile of a cohort of adults with DRFC on a battery of cognitive measures, in comparison to published age matched norms.
2. To investigate the demographic and clinical variables associated with cognitive dysfunction in individuals with DRFCs.

## Methodology

### Study design

A cross-sectional cohort study design was employed to investigate and characterise cognitive functioning in individuals with DRFCs.

### Ethical considerations

The research project was approved by the Melbourne Health Human Research Ethics Committee (Reference no. 62954/MH-2020) and was completed in accordance with the Helsinki Declaration.

### Participants

#### Recruitment

Participants were recruited from the Royal Melbourne Hospital Diabetic Foot Unit. This included inpatients, hospital outpatients, and community outpatients.

Participants were referred to the study by members of the participants’ treating team. Recruitment and data collection occurred between March 2021 and July 2022. During this time there were several COVID-19 related city-wide lockdown periods, and participants were seen via telehealth during this time.

#### Inclusion and exclusion criteria

Inclusion and exclusion criteria were designed to recruit an inclusive sample that is representative of the DRFC population treated in a public healthcare setting. Eligible participants were aged 18 years and above, diagnosed with either T1DM or T2DM and with a current DRFC or a DRFC within the past month. The eligibility of participants was determined by the researcher (MN), who is a psychologist and the clinical opinion of referring clinicians.

Individuals were excluded from the study if they were i) unable to provide informed consent, ii) not fluent in English iii) had hearing difficulties that precluded testing, or iv) had a severe medical, psychiatric and neurological disorders (e.g., delirium, advanced dementia, severe mood disorder, active psychotic disorder, active substance use disorder or other serious comorbid medical complications).

### Materials and measures

#### Sample characterisation and screening measures

Prior to participation, each individual completed a battery of screening measures, to determine eligibility and obtain demographic and general clinical information. These screening measures are presented in Table 1.

**Table 1:** Sample characterisation and screening measures

Measure	Domain Assessed	Description
The participant demographics questionnaire	Demographic information	This questionnaire was devised by the research team. This included demographic information such as gender, age, country of birth, English fluency, employment status and income and medical history
Alcohol Smoking and Substance Involvement Screening Test (ASSIST v3)	Substance use	This is an eight-item screening questionnaire developed by the World Health Organization to detect hazardous or harmful substance use [18]. Participants who endorsed use of any substance other than tobacco in the past three months were asked not to use the substance the night before research assessment
Visual Object and Space Perception Battery (VOSP) – Shape Detection Screening Test	Visual sensory capacity	This is a 20-item measure that asks participants to determine the presence of a degraded ‘X’ superimposed onto a scattered pattern. This task was assesses gross visual sensory capacity [19], and is scored as a pass or fail
Patient Health Questionnaire (PHQ-9)	Depressive symptoms	A 9-item self-report questionnaire which assesses the DSM-5 symptoms of Major Depressive Disorder [20]. This measure has been validated in DM populations [21]. This measure was included for the purposes of post-hoc analyses

##### ***Diabetes measures***

Information on DM type and duration, insulin use, and the presence of DM-related complications was obtained from medical records. Blood glucose was obtained using participants’ most recently recorded HbA1c level, which indicates average blood glucose level during the previous two to three months and long-term glycaemic control [22]. We employed a cut-off of 7.0% as an indicator of adequate glycaemic control, which is reflective of the general 7.0% HbA1C target generally recommended in DM [23, 24].

#### Diabetes-related foot complication measures

Classification of DRFC was according to the Society for Vascular Surgery Lower Extremity Threatened Limb (SVS WIfI) Classification System [25, 26]. This includes classifying the wound (size, depth, tissue loss and anticipated amputation/intervention requirements), ischaemia and foot infection. All foot measurements and classifications were made by podiatrists.

#### Cognitive assessment

All major cognitive domains were measured in the assessment battery and are described in Table 2. Measures were selected based on their relevance to the skills required for DRFC self-management, findings from previous research, their clinical utility, and ability to be administered over telehealth. To ensure feasibility, the proposed battery of measures was piloted with three members of the hospital’s Allied Health Consumer Representative Panel (without DRFC). Their feedback was discussed by the research team and adjustments were made accordingly. This included removal of cognitive measures that were considered to be overly time consuming or poorly tolerated by volunteering consumers.

**Table 2:** Cognitive assessment battery measures

Measure	Domain Assessed	Description
Advanced Clinical Solutions: * TOPF*	Premorbid Function	A 70-item list of words to provide an estimate of an individual’s highest level of cognitive ability, particularly when subsequent decline or impairment is suspected [27]. This task is scored based on the number of words correctly read, and has demonstrated very good reliability (*r* = .96-.99) and test re-test reliability (*r* = .89-.95), with age-matched norms [28]. Concurrent validity with the WAIS-IV Full Scale Intelligence Quotient has also been reported to be *r* = .70 [28]
CVLT-3-BF	Verbal Learning and Memory	Consists of a 9-word list presented over four trials, followed by short and long delayed recall and forced choice recognition [29]. It is a short-form of the widely used CVLT-3 for individuals with more severe cognitive deficits, used to reduce test burden[29]. Age-matched norms have been published by Delis et al. [29]. Across the CVLT-3 alternate, standard and brief forms, reliabilities ranging between *r* = .51 to .71 have been reported for the main scores capturing learning across trials, short and delayed recall [29]
Trail Making Test A	Psychomotor Speed	Requires participants to draw lines between sequential numbers as quickly as they can [30]. This is scored based on the amount of time taken to complete the task and has been found to have adequate test–retest reliability (*r* = .79) [31]. This study utilised norms as published by Tombaugh [30]
BVMT-R	Visual Learning and Memory	Requires participants to learn six line figures over three learning trials. This is followed by a delayed recall and recognition trial [32]. Reliability coefficients for the learning trials, total recall and delayed recall have been reported to be excellent (*r* = .96—.97), alongside age-matched norms[32]
Symbol Digit Modalities Test	Processing Speed	Participants are presented with unique symbol and digit pairings (Strober et al. 201) [33]. The oral version was used, requiring participants to state the corresponding number that is paired with each of a series of symbols as quickly as possible within 90 s. Scores measure the number of digits correctly read in 90-s. The SDMT has good concurrent validity with other commonly used measures of processing speed, ranging from *r* = 0.91 to *r* = 0.73 [34, 35]. Age-matched norms were as presented in Lezak et al. [36]
WAIS-IV: *Digit Span (Forwards and Backwards)*	Immediate Attention Span and Working Memory	In full, this is a three part verbal working memory task (reliability: *r*_*xx*_ = 0.93)1, [37] however only the first two parts were administered. The first requires participants to repeat a series of digits of increasing length, and in the second part participants are asked to recall digits in reverse order. This is scored based on the number of correctly repeated strings of digits. Normative data have been reported in Wechsler [38]
WAIS-IV: *Similarities*	Verbal abstract reasoning	Participants are asked to describe how two words (common objects or concepts) are similar, where scores reflect the quality of the answer and number of accurate responses provided before the discontinuation criterion is reached. This assesses verbal abstract reasoning and has a reliability of *r*_*xx.*_ = 0.871,[38]. Normative data have been reported in Wechsler [38]
D-KEFS: *Verbal Fluency*	Verbal Fluency and cognitive flexibility	The letter fluency component of this subtest requires participants to name as many words as possible beginning with a particular letter in 60 s, over three trials. The category fluency trials then ask participants to name items belonging to a semantic category over two trials. The category switching trial then requires participants to alternate between naming items from two different categories. Moderate split-half reliabilities have been established for letter fluency and category switching, ranging between .68 to .90 [39]. Age-matched normative data have been reported in Delis et al. [40]
The Hayling Sentence Completion Test	Inhibition control	This consists of two sets of 15 sentences, each with the last word missing. In the first set, participants are to complete the sentences, and measures response time. In the second set, sentences are to be completed with an unconnected word. This measures response inhibition, an aspect of executive function, and has normative data available [41]. In healthy adults, adequate test–retest reliability was found (*r* = .76). Modest correlations with other measures of executive function have also been reported (six elements test and the Tower of London; *r* = .40—.65; [42, 43]
BADS: Key Search Test	Planning and strategy generation	This is a subtest of the Behavioural Assessment of Dysexecutive Syndrome Battery with normative data available [44]. Participants are presented with a 100 mm square and asked to imagine that the square is a large field in which they have lost their keys and must draw a line to show where they would search the field to find their keys. Scoring is based on efficiency of the participants’ search strategy. The key search test has been found to have adequate test–retest reliability (*r* = .71) [45] and have significant correlation to another commonly used neuropsychological measure of planning and foresight (Porteus Maze Test) [46]

*TOPF* Test of Premorbid Function, *BVMT-R *Brief Visuospatial Memory Test, Revised, *WAIS-IV *Wechsler Adult Intellectual Scale, *CVLT-3-BF *California Verbal Learning Test, Third Edition—Brief Record Form, *DKEFS *Delis-Kaplan Executive Function System, *BADS *Behavioural Assessment of the Dysexecutive Syndrome

### Procedure

Participants undertook an assessment that was 60 to 90-min in duration. This was in the context of a larger study that also assessed psychological factors. The cognitive portion of the assessment was 50 to 60 min in length. Aside from the PHQ-9, psychological questionnaire data will be presented in a separate paper. Informed by the participants’ fatigue level, tolerance and personal preference, the cognitive and psychological assessment was completed across one or two sessions.

#### Telehealth assessment

Remote telehealth assessments (Zoom videoconferencing) were conducted during periods where COVID-19 restrictions applied. Face-to-face administration was resumed when deemed appropriate. Across all assessments, standardised test administration procedures were consistent.

#### Assessment structure

All assessments were conducted with the aim to administer measures in a consistent order. Variability in participants’ speed and fatigue levels led to minor changes in assessment structure, for some participants. Most assessments were conducted in the order as shown in Table 3.

**Table 3:** Typical assessment structure consisting of cognitive and screening measures

Order of Administration	Measure
1	ASSIST v3
2	VOSP Screen
3	CVLT 3 – Brief Version (Learning & Short Delay Trials)
4	BVMT-R (Learning Trials)*
5	BADS Key Search*
6	Trial Making Test – A*
7	WAIS-IV Digit Span (Backwards & Forwards)
8	CVLT 3 – Brief Version (Delayed Recall, Delayed Cued Recall & Recognition Trials)
9	WAIS-IV Similarities
10	Symbol Digit Modalities Test*
11	BVMT-R (Delayed Free Recall & Recognition Trials)*
12	Haylings Sentence Completion
13	DKEFS – Verbal Fluency
14	Test of Premorbid Function*
15	PHQ-9

*ASSIST v3 *Alcohol Smoking and Substance Involvement Screening Test, *VOSP *Visual Object and Space Perception Battery, *CVLT 3 *California Verbal Learning Test, Third Edition, *BVMT-R*, Brief Visuospatial Memory Test, Revised, *BADS *Behavioural Assessment of the Dysexecutive Syndrome, *WAIS-IV *Wechsler Adult Intellectual Scale, Fourth Edition, *PHQ-9 *Patient Health Questionnaire 9

^*^ Visual based task that may be excluded for participants with visual impairment

Due to the prevalence of visual impairment within the DRFC population [47], modifications to the research assessment were made for participants with visual impairments. Those who failed the VOSP Shape Detection Screening Test (score < 15) were not administered visually based tasks (refer to Table 3). Participants with visual impairment who passed the VOSP Shape Detection Screening Test (score > 15) were administered the complete assessment battery. However, to preserve the validity of each measure, individual measures were discontinued if the participant self-reported significant visual difficulty.

### Statistical analyses

#### Preliminary analyses and one-sample comparisons

Descriptive statistics were calculated for demographic variables. To compare performance on cognitive measures to age-matched population norms, one samples t tests were conducted for each measure. For data that did not meet normality assumptions, the most suitable transformation was applied (reflection and log transformations). No composite variables were used as all assessment tasks measured distinct and discrete cognitive functions.

#### Exploratory regression models

A series of hierarchical multiple regressions were undertaken to investigate the variables associated with cognitive performance. This was completed for each cognitive domain separately, using three predetermined consecutive models (Table 4). Independent variables in Model 1 consisted of participant demographic variables and assessment-related variables (e.g., whether all tests were completed by the participant). In an exploratory investigation, Model 2 included a wide range of diabetes-related clinical variables that were entered using stepwise regression. The stepping method criteria for this model was *p* = 0.05 for entry and *p* = 0.10 for removal. The criterion of *p* = 0.05 was utilised because of the exploratory nature of the study. This is in line with Rubin [48], where alpha-level adjustment is not required for exploratory analyses, as familywise error rates should consist only of different tests of the same hypothesis.

**Table 4:** Consecutive hierarchical models performed with method and predictors entered

Model	Method	Predictors
1	Enter	Age, gender, education, assessment completeness, assessment mode, patient setting type (inpatient/outpatient/community)
2	Stepwise	WIfI clinical stage, HbA1c, DM type, DM duration, ischemic heart disease, hyperlipidaemia, hypertension, congestive cardiac failure, retinopathy, nephropathy, macrovascular disease, neuropathy, overall number of diabetes complications
3 (Post-hoc)	Enter	Depression (PHQ-9)

Post hoc analyses were conducted for the purposes of sensitivity analyses. This involved conducting an additional hierarchical regression step (Model 3) with overall PHQ-9 score as a predictor in the final model.

## Results

Eighty participants were recruited from the Royal Melbourne Hospital Diabetic Foot Unit. Seventy completed the research assessment battery in full. Incomplete assessments were due to visual impairment (*n* = 7), inability to follow-up (*n* = 2) and fatigue (*n* = 1). An additional three participants consented to participate in the study but died prior to commencing the assessment. At the time of writing, eleven participants who completed the study had since died. Participant demographics and clinical characteristics are presented in Table 5.

**Table 5:** Participant demographics and clinical characteristics

Characteristic	Mean	SD (Range)	*n* (%)	*n* total
Age	63.38	11.44 (30 – 89)	-	80
Male	-	-	65 (86%)	80
Currently Employed	-	-	25 (31.3%)	80
Education				
< Highschool Completion	-	-	59 (73.8%)	80
Highschool Completion	-	-	17 (21.3%)	
Tertiary education	-	-	4 (5%)	
Patient Type				
Inpatient	-	-	29 (36.3%)	80
Hospital Outpatient	-	-	25 (31.3%)	
Community Outpatient	-	-	26 (32.5%)	
Type 2 Diabetes Type	-	-	71 (88.8%)	80
Vision Impaired	-	-	7 (8.8%)	80
Hba1c > 7%	-	-	56 (73.3%)	76*
Insulin Use	-	-	49 (61.3%)	80
Diabetes Complications				
Hypertension	-	-	53 (66.3%)	80
Ischaemic Heart Disease	-	-	28 (35%)	
Hyperlipidemia	-	-	37 (46.3%)	
Congestive Cardiac Failure	-	-	5 (6.3%)	
Macrovascular Disease	-	-	25 (31.3%)	
Retinopathy	-	-	15 (18.8%)	
Nephropathy	-	-	29 (36.3%)	
Neuropathy	-	-	50 (62.5%)	
Depressive Symptoms				
None – Minimal	-	-	36 (45.0%)	
Mild	-	-	18 (22.5%)	
Moderate	-	-	16 (20.0%)	
Moderately Severe	-	-	3 (3.8%)	
Severe	-	-	4 (5.0%)	
Assessment Mode				
Face to Face	-	-	72 (90%)	80
Telehealth	-	-	8 (10%)	
Assessment Completed in Full	-	-	70 (87.5%)	80

^*^Data unable to be obtained due to missing data in medical records

### One-Sample comparisons

The means and standard deviations for all neuropsychological measures are reported in Table 6. The differences between DRFC participant performance and expected performance levels was assessed by comparing against age-matched normative data where available, using one-sample *t* tests.

**Table 6:** Means for cognitive measures and comparisons against population means with effect sizes

Cognitive Domain	Cognitive Measure	Norms	M (SD)	*t (df)*	p-value	Cohen’s *d*
Premorbid Intellectual Functioning	Test of Premorbid Function	Holdnack and Drozdick (2009) [28]	95.4 (12.0)	-3.44 (79)	< .001*	-.385
Verbal Learning and Memory	California Verbal Learning Test	Delis et al. (2017) [29]				
	Learning trials index score		84.76 (20.12)	-6.80 (77)	< .001*	-.771
	Delay trials index score		81.19 (22.38)	-7.42 (77)	< .001*	-.841
Visual Learning and Memory	Brief Visuospatial Memory Test-Revised	Benedict (1996) [32]				
	Learning		49.92 (12.95)	-.054 (74)	.957	-
	Total Recall		43.17 (14.94)	-4.08 (74)	< .001*	-.471
	Delay		48.87 (17.95)	-.547 (74)	.586	-
Processing Speed	Symbol digit modalities test (oral)		-0.51 (1.32)	-3.22 (68)	< .001*	-.387
	WAIS-IV Digit Span	Wechsler (2009) [38]				
Immediate Attention	Forwards		9.56 (2.44)	-1.61 (78)	.111	-
Working Memory	Backwards		9.19 (2.50)	-2.88 (78)	.005*	-.824
Verbal Abstract Reasoning	WAIS-IV Similarities		7.72 (2.52)	-8.01 (77)	< .001*	-.907
Verbal Fluency	D-KEFS Verbal Fluency	Delis et al. (2001) [40]				
	Letter Fluency		8.75 (4.36)	-2.51 (76)	.014*	-.286
	Category Fluency		10.42 (3.73)	.976 (76)	.332	-
	Letter-Category Fluency Contrast		8.23 (3.93)	-3.94 (76)	< .001*	-.449
Set-shifting	Switching		9.03 (3.36)	-2.55 (76)	.006*	-.290
Inhibition Control	Haylings Sentence Completion	Burgess & Shallice, (1997) [41]	3.66 (2.06)	-9.55 (76)	< .001*	-1.146
Planning and Strategy Generation	BADS Key Search (z)		-.21 (1.11)	-1.64 (76)	.105	-

*WAIS-IV* Wechsler Adult Intellectual Scale, Fourth Edition, *D-KEFS *Delis-Kaplan Executive Function System, *BADS *Behavioural Assessment of the Dysexecutive Syndrome

^*^Significant following Benjamini–Hochberg correction

As shown in Table 6, standardised scores on TOPF (indicating premorbid intellectual functioning) were significantly lower in DRFC participants than the age-matched norms. However, it is noted that participants’ mean performance fell within the expected range (within 1 SD) of the mean performance of the age-matched normative data.

Across the cognitive domains, DRFC participants obtained significantly lower scores than age-matched norms. The greatest decrements (large effect sizes) were seen in inhibition control, verbal memory, verbal abstract reasoning, and working memory. Medium-sized decrements were seen in verbal learning and visual memory (total information recall during learning trials). Group performance decrements of small effect size were seen for set-shifting, letter category verbal fluency and processing speed.

Group performance on a planning and strategy generation task was consistent with population norms. However, there was a wide range of performance scores on this measure (*z*_Range_ = -2.73 – 4.09), with a portion of participants demonstrating considerable reductions in performance.

### Exploratory regression models

#### Learning and memory

Table 7 shows a series of multiple regressions explaining visual learning (total recall during BVMT-R learning trials) and memory (delayed recall). Both final models explaining the variance in scores on the BVMT-R had an overall large effect size. Higher HbA1c and the presence of hypertension were both associated with weaker visual learning. In addition, neuropathy was negatively associated with visual delayed recall, but retinopathy was associated with improved delayed recall.

**Table 7:** Multiple regression results for visual and verbal memory measures

Measure	Predictor		Model 1	Model 2	Model 3
BVMT-R Learning	Age	β	-.061	-.088	-.058
		*t*	-.508	-.754	-.518
		*sr*^*2*^	-	-	-
	Gender	β	-.085	-.116	-.124
		*t*	-.725	-1.013	-1.133
		*sr*^*2*^	-	-	-
	Education	β	.171	.213	.199
		*t*	1.425	1.814	1.757
		*sr*^*2*^	-	-	-
	Assessment Completeness	β	-.118	-.088	-.105
		*t*	-.941	-.715	-.889
		*sr*^*2*^	-	-	-
	Assessment Mode	β	.285	.282	.241
		*t*	2.398*	2.447*	2.149*
		*sr*^*2*^	.084	.089	.071
	Patient Setting	β	.049	.024	-.030
		*t*	. .387	.198	-.250
		*sr*^*2*^	-	-	-
	HbA1C	β		-.265	-.309
		*t*		-2.274*	-2.727*
		*sr*^*2*^		.078	.110
	Hypertension	β			-.278
		*t*			2.447*
		*sr*^*2*^			.091
BVMT-R Recall	Age	β	-.181	-.138	-.138
		*t*	-1.537	-1.201	-1.252
		*sr*^*2*^	-	-	-
	Gender	β	-.076	-.061	-.065
		*t*	-.650	-.543	-.601
		*sr*^*2*^	-	-	-
	Education	β	.065	.111	.163
		*t*	.541	.947	1.422
		*sr*^*2*^	-	-	-
	Assessment Completeness	β	-.286	-.279	-.290
		*t*	-2.321*	-2.344*	-2.536*
		*sr*^*2*^	.080	.082	.097
	Assessment Mode	β	.144	.124	.134
		*t*	1.213	1.081	1.221
		*sr*^*2*^	-	-	-
	Patient Setting	β	.093	.143	.178
		*t*	.749	1.175	1.516
		*sr*^*2*^	-	-	-
	Retinopathy	β		.278	.332
		*t*		2.367*	2.892*
		*sr*^*2*^		.084	.123
	Neuropathy	β			-.277
		*t*			-2.482*
		*sr*^*2*^			.093
CVLT 3 Brief Learning	Age	β	.046	.064	
		*t*	.409	.588	
		*sr*^*2*^	-	-	
	Gender	β	.224	.176	
		*t*	2.048*	1.633	
		*sr*^*2*^	.062	-	
	Education	β	.128	.098	
		*t*	1.124	.885	
		*sr*^*2*^	-	-	
	Assessment Completeness	β	-.374	-.343	
		*t*	-3.216**	-3.014*	
		*sr*^*2*^	.139	.126	
	Assessment Mode	β	.062	.062	
		*t*	.574	.569	
		*sr*^*2*^	-	-	
	Patient Setting	β	-.070	-.102	
		*t*	-.612	-.914	
		*sr*^*2*^		-	
	Macrovascular Complications	β		-.252	
		*t*		-2.261*	
		*sr*^*2*^		.075	
CVLT 3 Recall	Age	β	-.035		
		*t*	-.330		
		*sr*^*2*^	-		
	Gender	β	.229		
		*t*	2.191*		
		*sr*^*2*^	.070		
	Education	β	-.132		
		*t*	-1.212		
		*sr*^*2*^	-		
	Assessment Completeness	β	-.370		
		*t*	-3.325*		
		*sr*^*2*^	.147		
	Assessment Mode	β	.255		
		*t*	2.381*		
		*sr*^*2*^	.081		
	Patient Setting	β	.084		
		*t*	.770		
		*sr*^*2*^	-		

CVLT 3 = California Verbal Learning Test, Third Edition; BVMT-R = Brief Visuospatial Memory Test

^*^ p < *.05*

For verbal learning and memory, both multiple regression models explaining the variance in scores on the CVLT-3 had large effects sizes (Table 7). Female gender was associated with better learning of new verbal information and recall after a delay. The presence of macrovascular disease was significantly associated with weaker verbal learning. Interestingly, completing the assessment via telehealth mode was associated with higher scores on visual learning and verbal delayed recall, while premature assessment cessation was associated with lower scores on both visual recall and verbal learning and memory.

### Information processing

#### Psychomotor speed

Prior to analysis, TMT-A scores underwent reflection and log transformations to meet normality assumptions. On TMT-A, which assesses psychomotor speed, slower performance was associated the presence of ischaemic heart disease (Table 8). Longer diabetes duration and the presence of retinopathy was negatively associated with SDMT scores, which assesses information processing speed independent of psychomotor function (Table 8). Premature assessment cessation was also associated with slower scores on the SDMT. Both models had medium effect sizes.

**Table 8:** Multiple regression results for measures of attentional functions

Measure	Predictor		Model 1	Model 2	Model 3	Model 4
Symbol Digit Modalities Test	Age	β	-.176	-.125	-.141	
		*t*	-1.423	-1.056	-1.245	
		*sr*^*2*^	-	-	-	
	Gender	β	-.109	-.117	-.126	
		*t*	-.918	-1.041	-1.176	
		*sr*^*2*^	-	-	-	
	Education	β	.209	.208	.176	
		*t*	1.731	1.817	1.604	
		*sr*^*2*^	-	-	-	
	Assessment Completeness	β	-.272	-.261	-.295	
		*t*	-2.244*	-2.276*	-2.667*	
		*sr*^*2*^	.082	.089	.116	
	Assessment Mode	β	.155	.127	.148	
		*t*	1.282	1.112	1.345	
		*sr*^*2*^	-	-	-	
	Patient Setting	β	.096	.103	.047	
		*t*	.770	.879	.415	
		*sr*^*2*^	-	-	-	
	Diabetes Duration	β		-.315	-.320	
		*t*		-2.767*	-2.944*	
		*sr*^*2*^		.123	.138	
	Retinopathy	Β			-.278	
		*t*			-2.509*	
		*sr*^*2*^			.104	
Trail Making Test A	Age	β	.009	.001		
		*t*	.075	.005		
		*sr*^*2*^	-	-		
	Gender	β	.119	.073		
		*t*	.982	.620		
		*sr*^*2*^	-	-		
	Education	β	-.150	-.135		
		*t*	-1.212	-1.138		
		*sr*^*2*^	-	-		
	Assessment Completeness	β	.067	.081		
		*t*	.517	.653		
		*sr*^*2*^	-	-		
	Assessment Mode	β	-.120	-.178		
		*t*	-.981	-1.387		
		*sr*^*2*^	-	-		
	Patient Setting	β	-.250	-.178		
		*t*	-1.929	-1.387		
		*sr*^*2*^	-	-		
	Ischaemic Heart Disease	β		-.290		
		*t*		2.409*		
		*sr*^*2*^		.089		
WAIS-IV Digit Span (Forwards)	Age	β	-.037	-.043		
		*t*	-.313	-.371		
		*sr*^*2*^	-	-		
	Gender	β	.076	.068		
		*t*	.667	.604		
		*sr*^*2*^	-	-		
	Education	β	.240	.280		
		*t*	2.024*	2.381*		
		*sr*^*2*^	.059	.081		
	Assessment Completeness	β	-.220	-.235		
		*t*	-1.840	-2.011*		
		*sr*^*2*^	-	.060		
	Assessment Mode	β	.002	.014		
		*t*	.019	.125		
		*sr*^*2*^	-	-		
	Patient Setting Type	β	.085	.098		
		*t*	.707	.832		
		*sr*^*2*^	-	-		
	Neuropathy	β		-.235		
		*t*		-2.053*		
		*sr*^*2*^		.062		
WAIS-IV Digit Span (Backwards)	Age	β	-.013	.032	.027	-.026
		*t*	-.109	.284	.245	-.243
		*sr*^*2*^	-	-	-	-
	Gender	β	.099	.080	.071	. 061
		*t*	.889	.743	.680	.598
		*sr*^*2*^	-	-	-	-
	Education	β	.241	.242	.280	.296
		*t*	2.081*	2.174*	2.543*	2.790*
		*sr*^*2*^	.063	.069	.093	.112
	Assessment Completeness	β	-.160	-.143	-.157	-.157
		*t*	-1.370	-1.269	-1.430	-1.486
		*sr*^*2*^	-	-	-	-
	Assessment Mode	β	.227	.206	.217	.230
		*t*	1.986	1.870	2.022*	2.218
		*sr*^*2*^	-	-	.061	.073
	Patient Setting Type	β	-.208	-.204	-.192	-.140
		*t*	-1.771	-1.813	-1.746	-1.293
		*sr*^*2*^	-	-	-	-
	Diabetes Duration	β		-.274	-.278	-.278
		*t*		-2.513	-2.095*	-2.713*
		*sr*^*2*^		.090	.097	.106
	Neuropathy	β			-.224	-.257
		*t*			-2.095*	-2.468*
		*sr*^*2*^			.065	.089
	Congestive Cardiac Failure	β				.254
		*t*				2.418*
		*sr*^*2*^				.086

WAIS-IV = Wechsler Adult Intellectual Scale, Fourth Edition

^*^ p < *.05*

#### Immediate attention

A multiple regression predicting scores on the WAIS-IV Digit Span (Forwards) subtest found a positive association between scores on this measure and higher education and full assessment completion. The presence of neuropathy was associated with poorer performance on immediate attention. This final model was medium in effect size.

#### Working memory

A model with a large effect size found that higher education and congestive cardiac failure was associated with stronger working memory performance, as measured on the WAIS-IV Digit Span (Backwards) subtest (Table 8). The presence of neuropathy and longer DM duration were associated with weaker performance on this measure.

### Executive functions

#### Planning and strategy generation

Table 9 shows the results of the multiple linear regressions explaining planning and strategy generation skills on the BADS Key Search Test. The model had a large effect size. Higher HbA1c levels, and neuropathy were significantly associated with weaker performance on the BADS Key Search Test. Older age was also associated with poorer performance, yet it is noted that norms for this task were not adjusted for age.

**Table 9:** Multiple regression results for measures of executive function

Measure	Predictor		Model 1	Model 2	Model 3
BADS Key Search Test	Age	β	-.192	-.218	-.229
		*t*	-1.561	-1.955	-2.140*
		*sr*^*2*^	-	-	.069
	Gender	β	.021	-.025	-.028
		*t*	.176	-.229	-.264
		*sr*^*2*^	-	-	-
	Education	β	-.048	.020	.058
		*t*	-.386	.176	.525
		*sr*^*2*^	-	-	-
	Assessment Completeness	β	-.122	-.086	-.113
		*t*	-.945	-.733	-1.000
		*sr*^*2*^	-	-	-
	Assessment Mode	β	-.118	-.123	-.109
		*t*	-.956	-1.100	-1.015
		*sr*^*2*^	-	-	-
	Patient Setting	β	-.049	-.103	-.071
		*t*	-.380	-.871	-.626
		*sr*^*2*^	-	-	-
	HbA1c	β		-.439	-.380
		*t*		-3.914**	-3.453*
		*sr*^*2*^		.195	.162
	Neuropathy	β			-.277
		*t*			-2.511*
		*sr*^*2*^			.092
WAIS-IV Similarities	Age	β	.111		
		*t*	.977		
		*sr*^*2*^	-		
	Gender	β	-.027		
		*t*	-.249		
		*sr*^*2*^	-		
	Education	β	.357		
		*t*	3.136*		
		*sr*^*2*^	.133		
	Assessment Completeness	β	-.176		
		*t*	-1.527		
		*sr*^*2*^	-		
	Assessment Mode	β	.149		
		*t*	1.324		
		*sr*^*2*^	-		
	Patient Setting	β	-.138		
		*t*	-1.185		
		*sr*^*2*^	-		
Haylings Sentence Completion Test	Age	β	-.415		
		*t*	-3.614**		
		*sr*^*2*^	.171		
	Gender	β	.061		
		*t*	.547		
		*sr*^*2*^	-		
	Education	β	.146		
		*t*	1.278		
		*sr*^*2*^	-		
	Assessment Completeness	β	-.177		
		*t*	-1.543		
		*sr*^*2*^	-		
	Assessment Mode	β	.041		
		*t*	.357		
		*sr*^*2*^	-		
	Patient Setting	β	.135		
		*t*	1.156		
		*sr*^*2*^	-		
D-KEFS – Letter-Category Contrast Score	Age	β	.089	.065	
		*t*	.717	.539	
		*sr*^*2*^	-	-	
	Gender	β	.080	.070	
		*t*	.662	.605	
		*sr*^*2*^	-	-	
	Education	β	.222	.272	
		*t*	1.792	2.237*	
		*sr*^*2*^	-	.075	
	Assessment Completeness	β	.074	.059	
		*t*	.596	.495	
		*sr*^*2*^	-	-	
	Assessment Mode	β	.000	.014	
		*t*	-.001	.114	
		*sr*^*2*^	-	-	
	Patient Setting	β	.000	.170	
		*t*	1.134	1.379	
		*sr*^*2*^	-	-	
	Neuropathy	β		-.282	
		*t*		-2.370*	
		*sr*^*2*^		.083	
D-KEFS – Switching	Age	β	-.220		
		*t*	-1.800		
		*sr*^*2*^	-		
	Gender	β	-.015		
		*t*	-.129		
		*sr*^*2*^	-		
	Education	β	.093		
		*t*	.766		
		*sr*^*2*^	-		
	Assessment Completeness	β	-.253		
		*t*	-2.071		
		*sr*^*2*^	.064		
	Assessment Mode	β	-.011		
		*t*	-.090*		
		*sr*^*2*^	-		
	Patient Setting	β	.070		
		*t*	.565		
		*sr*^*2*^	-		

*BADS* Behavioural Assessment of the Dysexecutive Syndrome, *WAIS-IV *Wechsler Adult Intellectual Scale, Fourth Edition, *DKEFS *Delis-Kaplan Executive Function System

^*^ p < *.05*

^**^ p < *.001*

#### Verbal abstract reasoning

A multiple regression model explaining scores on the WAIS-IV Similarities subtest had a medium effect size (Table 9). Higher education was significantly associated with higher scores on this measure.

#### Inhibition control

The Hayling Sentence Completion test was explained by a multiple regression model with medium effect size (Table 9). Older age was found to be significantly associated with poorer scores on this measure but may also reflect the lack of age adjustment available within the norms for this measure.

#### Verbal fluency

Hierarchical multiple regression models for the DKEFS Verbal Fluency subtest including standardised performance scores for semantic category fluency (Appendix A, Table A1), letter-category fluency (Appendix A, Table A2), and contrast between Letter-category and semantic-category fluency (Table 9) were not statistically significant. However, the presence of neuropathy was significantly associated with a greater discrepancy between letter- and semantic-category fluency, with letter-category fluency being weaker.

#### Set shifting

Multiple regression models for DKEFS Verbal Fluency Subtest (Switching) were not statistically significant (Table 9). Nevertheless, when all other variables were controlled, telehealth assessment mode was associated with lower scores.

### Post-hoc analyses

 Across all regression models predicting cognitive measures, PHQ-9, a measure of depressive symptoms, in the model yielded no statistically significant change (p > 0.05), and therefore was not included in final models.

## Discussion

Our study aimed to characterise cognitive functioning and identify the demographic and clinical predictors of cognitive performance in individuals with DRFC, with a study design that closely parallels clinical neuropsychologists’ practice through the use of common neuropsychological measures and age-matched norms. To our knowledge, this is the first study to employ a comprehensive battery of neuropsychological measures within a DRFC cohort. Participants were predominantly male and demonstrated significantly weaker performances relative to age-matched norms in a range of cognitive domains, with reductions generally moderate to large in effect size. However, the cohort also scored significantly lower on a task evaluating premorbid cognitive functioning, and had lower educational attainment, with the majority not completing high school. On exploratory analysis, several demographic and diabetes factors, such as vascular disease, also appear to be associated with poorer cognitive function.

### Cognitive performance

Our findings highlight reduced cognitive functioning in verbal memory, attention, processing speed and executive functioning in DRFC*.* This aligns with existing literature identifying reduced cognitive functioning in both the general DM and DRFC population, where global cognitive dysfunction has been reported [4, 8, 49]. Notably, however, when compared to the effect sizes found in a meta-analysis by Palta et al. [4] comparing T2DM samples to non-diabetic controls, the reductions in performance in the current DRFC sample are considerably larger. Therefore, on similar neuropsychological measures and domains, individuals with DRFC appear to show weaker performance than those from general T2DM cohorts. However, in contrast to the comparison studies, age-matched normative data acted as the comparison measure in the current study, rather than a study-matched control group. This may, to some degree, have contributed to the larger effect sizes. Against the most comprehensive previous study examining cognition in DRFC [8], our findings were mostly consistent. However, Natovich et al. [8] did not employ measures of visual memory and undertook only broad domain-level analyses. Furthermore, the study used a computerised battery that had not been broadly investigated in DM literature or used commonly in clinical practice. The current study used well-researched neuropsychological measures commonly used in clinical practice [50]. In addition, by using neuropsychological measures that are sensitive to more specific cognitive functions with analyses at the subtest level, we found more consistent verbal memory reductions relative to visual memory in DRFC, with visual recall an area of comparatively preserved cognitive skills. This could have significant implications for self-management interventions, which could focus on visual memory aids and prompts using pictures and diagrams rather than words.

Nevertheless, there were some domains that appeared preserved. No significant differences were found between population norms and the DRFC cohort on measures of immediate attention or planning and strategy generation. However, there was a wide variance in performance across the sample in planning and strategy generation, where a portion of the participants demonstrated a considerably reduced performance on this measure.

### Exploratory analyses: Factors associated with poorer cognitive function

#### Participant and demographic factors

Understanding the factors that influence the magnitude of reduction in cognitive functioning in individuals with DRFC is also vital. We found that higher education was associated with better performance on verbal abstract reasoning, verbal fluency, working memory and immediate attention. This corresponds with existing literature in both general and DM populations [51, 52]. In particular, education may influence the development of cognitive skills and improve cognitive reserve, acting as a protective factor in older age, leading to stronger cognitive functioning [53, 54]. Female gender was also associated with stronger verbal memory. This is consistent with previous reports of superior verbal memory in females than males [55] and has been theorised to be due to both psychological factors and biological factors, such as increased hippocampal volume, availability of dopamine transporters and estrogen-induced synaptic changes [55–58]. As an important caveat, our findings are limited by an uneven gender distribution within the sample (85% male), a distribution commonly seen in DRFC studies [10]. Further, as previously discussed by Fisher [59], it is not possible to determine whether similar participant factors impacted memory functioning in a previous study with a DRFC cohort [8], as it is not clear what form of memory measure was utilised. Notably, depressive symptoms did not influence the relationship between cognitive impairment and any participant or clinical factors, suggesting that the cognitive difficulties identified cannot be attributed to depression.

#### Diabetes and clinical factors

##### Glycaemic Control

A number of clinical factors were also associated with cognitive performance. Our study found poorer glycaemic control (HbA1c) was negatively associated with visual memory (learning), planning and strategy generation. Similar findings in T1DM and T2DM cognition have been reported, but have not yet been investigated in a DRFC cohort [60, 61]. Our findings emphasise the importance of rigorous metabolic control in DRFC, and the need for effective management of cognitive problems to minimise further decline in cognition. The critical nature of this issue can be seen by the number of participants who died by the time of writing (approximately one to two years following participation), which is concurrent with established mortality rates [2]. Of those who died (*n* = 11), 91% met the criteria for Mild Cognitive Impairment (MCI) at the time of participation, potentially reflecting an emerging dementia. This was defined by scores more than 1.5 standard deviations below normative expectations, in at least one cognitive domain, as typically seen in MCI literature [62–64]. This finding points towards potentially even more severe disease and mortality in those with concurrent DRFC and MCI. This aligns with research demonstrating higher mortality rates in patients with comorbid MCI and cardiovascular disease [65]. It highlights the need for cognitive screening to identify individuals at increased risk, who may need prioritised support*.* Further investigation of this link between dementia and diabetes in older adults may be warranted.

##### Macrovascular Disease

Macrovascular disease is also common in DM and raises the risk of cerebral ischaemia, stroke and cognitive impairment [66–69]. Supporting this, we found that the presence of one or more macrovascular complications (i.e., stroke, ischaemic heart disease, congestive cardiac failure, peripheral artery disease) was associated with poorer verbal learning, but was not associated with poorer performance in any other measured cognitive domains. Ischaemic heart disease, specifically, was also associated with weaker performance on a processing speed task. This is comparable to previous studies reporting cognitive impairments following coronary events [70], atrial fibrillation and stroke [71], by way of abnormal cardiac output [72]. Similar findings have also been replicated in the general DM population [73], but general macrovascular complications have not been widely investigated cross-sectionally in DM with or without foot complications. Interestingly, however, congestive cardiac failure was associated with better working memory. This is an unexpected finding and may have been impacted by the small sample size of participants with this complication. It is noted that cognitive impairment has been previously established within this population, including in working memory [74], so this finding is most likely a statistical anomaly due to sampling error within the small cohort (*n* = 5). Further clarification of this relationship in a larger sample would be beneficial.

##### Microvascular Disease

Changes in microvasculature can also reduce cerebral blood flow and contribute to stroke and dementias [75–78]. Retinopathy was associated with slower processing speed, which is not overly surprising, given the visual component in this task. Wu et al. [79] reported that diabetic retinopathy was associated with deficits in psychomotor and attentional functions. We did not find an association between reductions in psychomotor speed and retinopathy, potentially due to greater levels of overall psychomotor slowing in our cohort. However, we interestingly found an association between retinopathy and better visual delayed recall. Again, this is most likely to be a spurious finding. A sampling error is probable due to the small sample size with retinopathy (*n* = 15), and in light of existing literature, including meta-analyses, that have established links between DM retinopathy and poorer cognitive function [80, 81]. Future investigation in a larger sample may be useful to clarify this. In addition, neuropathy was negatively associated with visual memory recall, immediate attention, working memory and verbal fluency. This is consistent with previous findings in general T1DM and T2DM cohorts, where peripheral neuropathy has been previously associated with general cognitive impairment [82], due to somatosensory and motor difficulties impacting on test performance. Such difficulties may have contributed to the reduced scoring on the BVMT-R in our study, but other associated measures did not require any form of motor involvement (i.e., were verbally based). Therefore, this may instead represent underlying changes in cerebral microvasculature. Overall, it appears likely that vascular complications alongside DRFC may be a harbinger for reduced cognitive function in certain domains.

##### Diabetes Duration and Foot Complication Severity

In addition to vascular complications, longer diabetes duration was also associated with weaker information processing speed and working memory. This link does not appear to have been described in DRFC previously but is consistent with findings in both T1DM and T2DM [83, 84]. Interestingly, DRFC severity (WIfi clinical stage) and patient setting were not associated with differences cognitive functioning. To our knowledge, this is the first investigation of the relationship between cognitive functioning and DRFC severity, and points towards the stable nature of cognitive problems, which may not vary substantially according to severity of the foot-related complication. That is, just the presence of foot-complications, regardless of their severity, is sufficient to act as a risk factor, or correlating factor, of cognitive dysfunction. Further longitudinal investigation of these relationships would be beneficial, that go beyond the point-prevalence snapshot obtained in the current study.

##### Limitations

Our findings should be considered within the context of certain limitations. While there were minimal exclusion criteria, there remains subsets of the DRFC population that were not adequately represented. Due to the requirements for English fluency, non-English speaking patients were excluded from the study. Patients who declined to participate in our study may have also been a subset of the population who potentially had lower health literacy, insight into cognitive difficulties or higher anxiety or depression.

Further impacting the generalisability of our study is the considerable gender imbalance within the sample cohort (86% male). Therefore, the generalisability of the results to female DRFC patients is unclear. Female gender appears to be associated with less severe DRFC disease in previous literature [85, 86] and potentially better cognitive function. This is supported by our exploratory finding of better verbal memory in female participants. Nevertheless, our sample is reflective of the wider DRFC population that is predominantly male, and therefore provides useful insight into the general DRFC population [87]. To fine-tune understanding of gender differences within DRFC, future investigations in DRFC cohorts with more female participants would be beneficial to inform targeted treatment approaches, particularly in light of existing evidence of differential treatment outcomes.

In addition, as a consequence of the COVID-19 pandemic, a number of participants were required to complete the assessment battery via telehealth (*n* = 8). To account for this, the assessment mode was controlled for in multiple regression analyses. Nevertheless, telehealth administration was significantly associated with better performance on two memory measures. Instead of reflecting differences in testing procedures, this likely indicates a degree of higher cognitive capability in those who are able to operate telehealth technology*.* Computer use has been associated with better cognitive function across adulthood [88]. The ability to navigate technology requires a certain level of cognitive function, including memory. Individuals who are more severely unwell or with lower cognitive function may be more likely to face challenges in using telehealth, therefore potentially leading to sampling bias*.* Nevertheless, cognitive measures in this study were selected for their clinical utility and robust telehealth psychometric properties [89]. In particular, the administration of verbally mediated tasks by qualified professionals have been reported to not be affected by videoconference administration [89], and therefore can reliably measure their relevant construct via telehealth. Replication of our findings using the same test procedures across all participants would nevertheless be beneficial.

Therefore, while our study provides valuable insights into cognitive function in DRFC, the aforementioned limitations of the sample highlight the importance of interpreting and generalising these findings with caution. It is possible, for example, that individuals who have lower health literacy or higher anxiety or depressive symptoms, may perform poorer on cognitive measures, as a consequence of the influences of education, mood and anxiety on cognition [61–63]. Inadequate representation of comorbidities (i.e. retinopathy, congestive cardiac failure) may also reduce generalisability due to sampling error. Future research including larger and broader DRFC samples, with sufficient inclusion of female participants, those with other comorbid DM complications, and with matched participant levels of technological proficiency, may be useful to determine the generalisability of our results. Our study makes comparisons between DRFC test performance and age-matched population norms. It is recognised that this limits the strength of the conclusions that can be drawn from findings. Direct comparisons between a control group that are similar in sex, education, economic and cultural background would have allowed for clearer conclusions about cognitive function in DRFC. Recruitment of a control group with DM patients without foot complications was initially intended, however, due to COVID-19 infection control procedures, there was limited access to this group. Further research involving direct comparisons with a non-foot complication control group would be useful to determine if similar results are found with a study-matched control sample.

Finally, given the exploratory nature of this research, there were a large number of variables analysed in relation to the sample size. As according to Thayer [90] and Tredennick et al. [91] we have approached the analyses with a broad exploratory research question which has aimed to identify the “best” from a set of plausible variables. Our exploratory findings will be useful to guide future targeted research.

##### Practical implications

Addressing cognitive dysfunction in the DRFC population is likely to assist in strengthening the efficacy of DRFC self-management and treatment success. Based on our findings, clinicians are recommended to utilise a range of strategies to compensate for cognitive difficulties. This includes visual aids and notes to take advantage of the relative strength in visual memory compared to verbal memory. Compensating for weaknesses in executive functions, working memory and processing speed may involve minimising the volume of information provided at each session, removing distractions (e.g., limiting the number of people in a room) and utilising simple and literal language when providing education. Environmental modifications within DRFC patients’ homes that support areas of cognitive difficulty may also be useful to promote ideal self-management behaviours. Thorough assessment of patients’ risk factors (higher HbA1c levels, significant macrovascular and microvascular disease) may also identify individuals who would benefit from further assessment and cognitive support. Routine cognitive screening within this patient group, with potential for referral for more comprehensive neuropsychological assessment, is also recommended given elevated risk of reduced cognitive function. A full cognitive profile could then be used to design individually specific and tailored cognitive strategies, based on the unique cognitive profile of the patient. These findings can also be used to inform future research, including longitudinal study designs and the design of a cognitive intervention program to optimise self-management. Such programs have been used successfully in other clinical populations where cognitive impairment impacts health behaviours [92].

## Conclusions

Our study provides a comprehensive characterisation of cognitive function in individuals with DRFC and is unique in its applicability to be translated into clinical practice. In particular, our use of commonly used neuropsychological measures and comparisons with age-matched norms closely reflects the typical practice of clinical neuropsychologists. Our findings demonstrate fairly generalised global cognitive dysfunction relative to age-matched norms within this patient group, with reductions in verbal memory and executive functioning being the most prominent. We have also found associations between education, gender and indicators of diabetes severity and cognitive function. Diabetes-related and vascular complications are also associated with more pronounced cognitive dysfunction. Given the exploratory nature of these analyses, further research to clarify these relationships between demographic and clinical variables with cognition is warranted in a larger sample. As DRFCs are a serious complication that have devastating outcomes if not successfully managed, it is imperative that treatment and self-management is optimised by addressing cognitive problems. Further comprehensive explication of the relationships between DM complications and cognitive function in DRFC in a longitudinal manner would be most useful to inform delivery of care.

### Supplementary Information

Below is the link to the electronic supplementary material.

Supplementary file1 (DOCX 14 KB)

## Data Availability

The data that support the findings of this study are available from the corresponding author, CF, upon reasonable request.
