# Supplementary material for: Cognitive dysfunction in diabetes-related foot complications: A cohort study
Source: J Diabetes Metab Disord. 2024 Jan 22;23(1):1017–38. doi: 10.1007/s40200-023-01381-4 (PMC11196439; doi:10.1007/s40200-023-01381-4)
Supplement: Supplementary file 1 — Supplementary file1 (DOCX 14 KB) [file 40200_2023_1381_MOESM1_ESM.docx]

**Appendix A**

***Table A1.***

Results of multiple linear regression models assessing predictors of semantic fluency

| Predictor | Model 1  R square = .122  *p* = .206 | |
| --- | --- | --- |
|  | β | *t* |
| Age | -.193 | -1.574 |
| Gender | .140 | 1.176 |
| Education | .033 | .269 |
| Assessment Completeness | -.132 | -1.071 |
| Assessment Mode | .202 | 1.663 |
| Patient Type | -.077 | -.615 |

***Table A2.***

Results of multiple linear regression models assessing predictors of phonemic fluency

| Predictor | Model 1  R square = .139  *p* = .138 | |
| --- | --- | --- |
|  | β | *t* |
| Age | -.089 | -.733 |
| Gender | .183 | 1.552 |
| Education | .203 | 1.665 |
| Assessment Completeness | -.071 | -.586 |
| Assessment Mode | .186 | 1.545 |
| Patient Type | .020 | .165 |
